# Liraglutide 3.0 mg (Saxenda©) for Weight Loss and Remission of Pre-Diabetes. Real-World Clinical Evaluation of Effectiveness among Patients Awaiting Bariatric Surgery

**DOI:** 10.1007/s11695-023-06895-7

**Published:** 2023-11-28

**Authors:** Rebekah Wilmington, Arash Ardavani, Amelia Simenacz, Carol Green, Iskandar Idris

**Affiliations:** 1 East Midlands Bariatric Metabolic Institute, University Hospitals of Derby and Burton NHS Foundation Trust, Derby, DE223DT UK; 2 Centre of Metabolism, Ageing & Physiology, Nottingham, NIHR BRC, Division of Medical Sciences and Graduate Entry Medicine, School of Medicine, Royal Derby Hospital, University of Nottingham, Uttoxeter Road, Derby, DE223DT UK; 3 University of Nottingham, Medical School, Nottingham, NG72UH UK

**Keywords:** Tier 3 service, Tier 4 service, Liraglutide, Prediabetes, Glycaemic control, Obesity management

## Abstract

**Objective:**

The effectiveness of liraglutide 3.0 mg (Saxenda) therapy to induce weight loss among obese patients prior to bariatric surgery remains uncertain.

**Methods:**

Clinical data was retrospectively obtained from patients with prediabetes (HbA1c 42-47 mmol/mol) and selected patients on the waiting list for bariatric surgery at the Royal Derby Hospital. Clinical data was collected retrospectively at 6, 12, 26 and 52 week intervals. The outcomes included mean weight change, proportion of patients achieving ≥ 5% and ≥ 10% weight loss and achieving HbA1c reduction to normal range values.

**Results:**

Fifty patients (mean age of 46.2 ± 10.5 years; 76% female and 94% had Class III obesity) who completed 52 and/or 26 weeks of treatment were included. Liraglutide 3.0 mg produced a consistent and statistically significant reduction in weight (kg), BMI (kg/m2) and HbA1c (mmol/mol) across all four time intervals. Average ± SD reduction for weight, BMI and HbA1c respectively at 26 weeks were: -10.9 ± 9.1 (*P *< 0.01), -3.67 ± 3.5 (*P *< 0.01), -4.7 IQR 4.95 (*P* < 0.001), and at 52 weeks were: -14 ± 9.2 kg (*P* < 0.001), -4.64 ± 4.0 (*P* < 0.001 and -5.5 IQR 4 (*P* = 0.009). 85.7% and 33.3% of patients achieved ≥ 5% and 10% weight loss target respectively at 52 weeks. 92.3% and 72.2% achieved remission of pre-diabetes by 6 and 12 months respectively. Liraglutide 3.0 mg was well-tolerated with only 10% discontinuing medication due to tolerability issues.

**Conclusion:**

Liraglutide 3.0 mg, with lifestyle management, reduced weight and improved glycaemic control. These results support liraglutide’s application in certain high-risk populations, including patients waiting for bariatric surgical intervention.

Glucagon-like peptide-1 analogues such as liraglutide 3.0 mg (Saxenda) is an effective therapy to induce weight loss in clinical trials [1, 2] when combined with lifestyle interventions but its effectiveness among obese patients prior to bariatric surgery remains uncertain [3]. We undertook service evaluation of the effectiveness of Saxenda© on weight loss and glycaemic control, including remission rate from pre-diabetes state among obese patients attending a specialist bariatric service [4].

## Methods

We included both Tier 3 patients with a diagnosis of prediabetes (HbA1c 42–47 mmol/mol) and Tier 4 patients on the waiting list for bariatric surgery since 2019 at Royal Derby Hospital, a regional centre for bariatric surgery across the East Midlands. The planned surgeries for this patient cohort included both Roux-en-Y bypasses and Sleeve Gastrectomies. The waiting times for surgery reflect pressures to the bariatric services across the UK, inclusive of prior COVID-19 delay. Our criteria for referral for bariatric surgery is based on the UK National Institute of Clinical Excellence (NICE) guidelines which stipulate that adults with a body mass index (BMI) of 40 kg/m^2^ or more, or between 35 kg/m^2^ and 39.9 kg/m^2^ with a significant health condition that could be improved if they lost weight, can be referred for bariatric surgery assessment [5].

All Saxenda © treatment was initiated and delivered as per current NICE guidelines [6], in brief recommended for patients with obesity, non-diabetic hyperglycaemic and a high risk of cardiovascular disease whilst licensed as an adjunct for weight loss alongside a hypocaloric diet and increased physical activity. Weight management services are delivered in tiers: Tier 1 is universal e.g. health promotion, Tier 2 are delivered by local community services and focus on lifestyle interventions, Tier 3 is a specialist service delivered by clinician-led multidisciplinary team typically providing an integrated non-surgical approach, Tier 4 covers those patients proceeding to bariatric surgery.

Eligible patients for Saxenda© were discussed at the Tier 3 multi-disciplinary team meeting (MDT) and were then counselled by a Tier 3 clinician to consent for commencement of Saxenda©. The programme to initiate Saxenda© was supported by the Oviva Patients Support group commissioned by Novo Nordisk who provided patient education regarding medication administration, troubleshooting advice and further details. Thereafter, patients received regular follow-up with the Tier 3 clinician assuming their ongoing compliance and ready engagement with the service.

Clinical data was collected retrospectively at 6, 12, 26 and 52 week intervals. The outcomes included mean weight change, proportion of patients achieving ≥ 5% and ≥ 10% weight loss and achieving HbA1c reduction to normal range values.

This is an ongoing clinical service. Data evaluated was available for 50 patients who had either reached the 26 week point and/or 52 week point. The number reflects those at these timepoints who had been found eligible at corresponding MDT discussion. This was from a total cohort of 83 patients who had been initiated on Saxenda©. Patients received liraglutide 3.0 mg treatment between 9^th^ January 2021 and 28^th^ May 2023. Four variables of interest from baseline: age (years), HbA1c (mmol/mol), weight (kg) and BMI (Kg/m^2^), were all tested for distributional normality using the Kolmogorov–Smirnov test and histogram visual inspection. Where available, the delta (Δ) for all available individual data per variable of interest from baseline (0 weeks) with the 6-, 12-, 26- and 52-week follow-up intervals was assessed. An 83 patient cohort was identified, a total of 50 patients had data available for the 6 week, 12 week and 26 week time intervals, and within these 50 patients, 22 had proceeded for a 12 monthly review.

### Statistical Analysis

Student’s paired t-test and the Wilcoxon signed rank test were selected to assess the statistical significance of the variables of interest, with the significance threshold predetermined to α = 0.05 (2-tailed). Participant entries containing missing data were excluded from each relevant interval of analysis. Formal statistical assessments and graphical outputs were undertaken using IBM SPSS Statistics v18.0.1.1.

## Results

From a cohort of 83 patients, 50 patients (mean age of 46.2 ± 10.5 years; 76% were female and 94% had Class III obesity) who completed 52 and/or 26 weeks of treatment were included. (Table 1). Saxenda© produced a consistent and statistically significant reduction in weight (kg), BMI (kg/m2) and HbA1c (mmol/mol) across all four time intervals (6-,12-,26- and 52 weeks). average ± SD reduction for weight, BMI and HbA1c respectively at 26 weeks were: -10.9 ± 9.1 (*P* < 0.01), -3.67 ± 3.5 (*P* < 0.01), -4.7 IQR 4.95 (*P* < 0.001), and at 52 weeks were: -14 ± 9.2 kg (*P* < 0.001), -4.64 ± 4.0 (*P* < 0.001 and -5.5 + 4 (P = 0.009) [Table 2]. 85.7% of patients achieved and maintained their ≥ 5% weight loss target at 52 weeks, 33.3% achieved a ≥ 10% weight loss and 9.5% saw a weight loss of ≥ 15% in the same time period.

**Table 1:** Baseline characteristics of cohort

Characteristics	N	Mean	SD	Range
Age (years)	50	46.2	10.5	26 to 72
Baseline weight (kg)	50	153.8	29.3	105 to 226
Body mass Index (kg/m^2^)	50	54.1	10.1	37 to 79.8
Characteristics	N	Median	IQR	
HbA1c (mmol/mol)	50	43	42.0 to 44.2	
Characteristics	N	Proportion (%)		
Gender (Female %)	38	76		
Class 1 Obese	0	0		
Class 2 Obese	3	0		
Class 3 Obese	47	94

**Table 2:** Tabulated results for weight, BMI and HbA1c

	WEIGHT (kg)			Body mass Index (kg/m^2^)			HbA1c (mmol/mol)		
	Mean difference	SD	*P*-value	Mean difference	SD	*p*-value	Median	IQR	P-value
0 v 6 wks	-4.82	5.7	< 0.001*	-1.56	2.6	< 0.001*			
0 v 12 wks	-7.36	23.9	0.043	-2.54	3.4	< 0.001*	3.0	4.0	0.002*
0 v 26 wks	-10.9	9.1	< 0.001*	-3.67	3.5	< 0.001*	4.7	4.5	< 0.001*
0 v 52 wks	-14.0	9.2	< 0.001*	-4.64	4.0	< 0.001*	5.5	4.95	0.009*

By 3 months, 71.8% had their HbA1c return to normal (< 42 mmol/mol) with an average HbA1c value of 40.65 mmol/mol. Similarly, 92.3% achieved this remission by 6 months with an average HbA1c of 38.46 and 72.2% achieved this remission at 12 months with an average HbA1c of 37.7%.

**Fig. 1:**
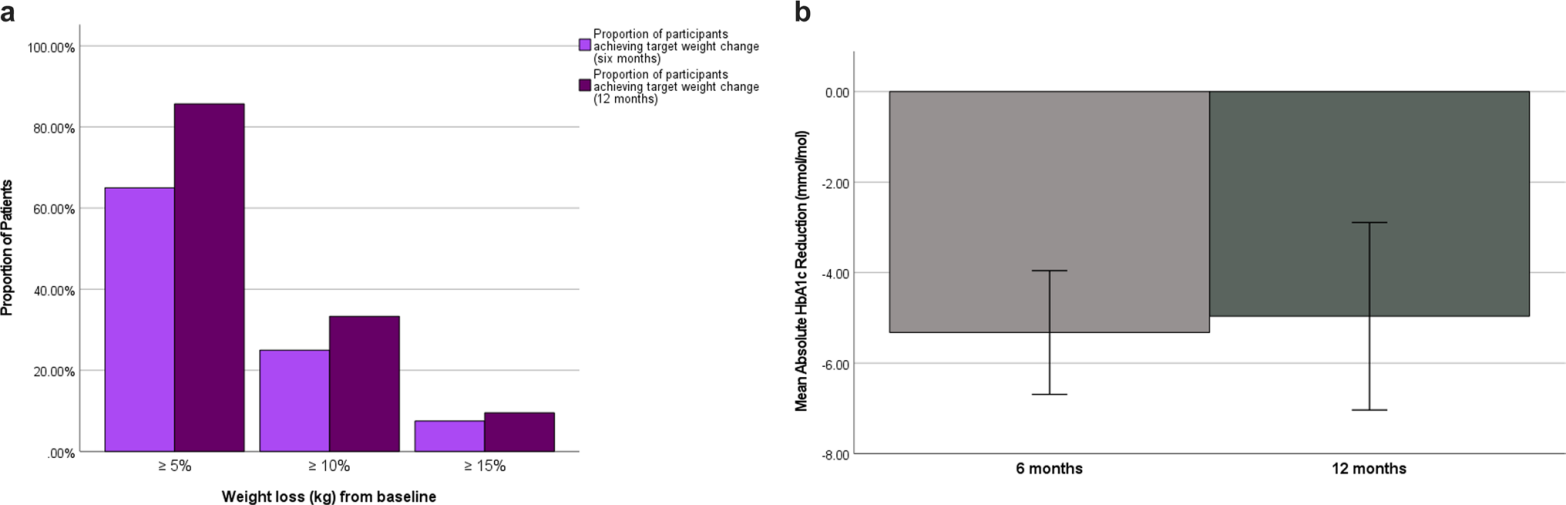
(**a**) Proportion of patients achieving > 5%, > 10% and > 15% weight reduction at 26- and 52-weeks; (**b**) Absolute HbA1c decrease observed at 26-weeks and 52 weeks following liraglutide 3.0 mg treatment. Error bars 95% CI

Saxenda© was well-tolerated with the maximal tolerated dose being 2.84 ± 0.43 mg and only 10% discontinuing medication due to tolerability issues. Reported side effects included nausea (22.0%), abdominal pain (4.0%), constipation (6.0%), sulphurous eructation (14%), headache (10%), reduced appetite (12%), diarrhoea (10.0%), vomiting (8.0%), dry mouth (4.0%). Overall 15 of the 50 patients discontinued treatment with Saxenda©: 80% of these discontinuations were due to external reasons e.g. being listed for bariatric surgery, 6.7% due to compliance issues and 26.7% due to symptoms/tolerability (Fig. 1).

## Conclusions

Saxenda© 3.0 mg, alongside a specialist weight-management service, consistently demonstrates significant reduction of weight, reduced HbA1c and reduced progression to diabetes over one year, while also well tolerated for obese patients awaiting bariatric surgical intervention [7].

## Data Availability

Data is available within reasonable request to the corresponding authors.
